# Convolutional Neural Network Addresses the Confounding Impact of CT Reconstruction Kernels on Radiomics Studies

**DOI:** 10.3390/tomography7040074

**Published:** 2021-12-03

**Authors:** Jin H. Yoon, Shawn H. Sun, Manjun Xiao, Hao Yang, Lin Lu, Yajun Li, Lawrence H. Schwartz, Binsheng Zhao

**Affiliations:** 1 Department of Radiology, New York Presbyterian Hospital, Columbia University Irving Medical Center, New York, NY 10039, USA; jhy2118@cumc.columbia.edu (J.H.Y.); shawnsun25@gmail.com (S.H.S.); yh2588@cumc.columbia.edu (H.Y.); lhs2120@cumc.columbia.edu (L.H.S.); bz2166@cumc.columbia.edu (B.Z.); 2 Department of Radiology, The Second Xiangya Hospital, Central South University, Changsha 410011, China; xiaomanjun@csu.edu.cn

**Keywords:** radiomics, reproducibility, convolutional neural network, computed tomography, kernel conversion, quantitative imaging

## Abstract

Achieving high feature reproducibility while preserving biological information is one of the main challenges for the generalizability of current radiomics studies. Non-clinical imaging variables, such as reconstruction kernels, have shown to significantly impact radiomics features. In this study, we retrain an open-source convolutional neural network (CNN) to harmonize computerized tomography (CT) images with various reconstruction kernels to improve feature reproducibility and radiomic model performance using epidermal growth factor receptor (EGFR) mutation prediction in lung cancer as a paradigm. In the training phase, the CNN was retrained and tested on 32 lung cancer patients’ CT images between two different groups of reconstruction kernels (smooth and sharp). In the validation phase, the retrained CNN was validated on an external cohort of 223 lung cancer patients’ CT images acquired using different CT scanners and kernels. The results showed that the retrained CNN could be successfully applied to external datasets with different CT scanner parameters, and harmonization of reconstruction kernels from sharp to smooth could significantly improve the performance of radiomics model in predicting EGFR mutation status in lung cancer. In conclusion, the CNN based method showed great potential in improving feature reproducibility and generalizability by harmonizing medical images with heterogeneous reconstruction kernels.

## 1. Introduction

Radiomics has emerged as a potential aid to non-invasively characterize tumors using images [1,2,3,4]. Radiomics extracts quantitative features from medical images that can describe lesion characteristics in detail, thus completing and supporting the radiologist visual assessment. These quantitative features are then used to build models that can provide valuable clinical information to direct patient treatment. Multiple studies have shown that radiomics can aid in predicting cancer prognosis [5,6,7,8], a tumor’s gene mutation status [9,10,11], and tumor recurrence [1,12,13,14]. However, current radiomics studies are limited in their ability to use large, multi-center data because heterogeneous computerized tomography (CT) acquisition parameters can be confounding factors [15].

The literature shows that CT scanners, scanning techniques, reconstruction parameters, and other non-clinical variables can alter the computed feature values in radiomics studies and thus influence the conclusions of these studies. A recent article comprehensively reviewed sources of variations and potential strategies to reduce such variations in radiomics [16]. In order to compare and conduct multi-center studies and to improve the generalizability of radiomic results, various techniques have been proposed: controlling image acquisition parameters, processing images (e.g., resampling images, filtering the images) post image acquisition and prior to feature extraction, converting images to a desired imaging setting, standardizing the definitions of features, and harmonizing feature values statistically using the ComBat method [17,18,19,20,21,22,23,24,25,26,27]. Although there are many methods being investigated to improve radiomics research, it is difficult to assess which one is better. There are no published direct comparisons, and Mali et al. [28] and Ibrahim et al. [29] recently published review articles in which they both discuss the need for further investigations on harmonization methods to analyze radiomics data using the available retrospective and unpaired imaging data from multiple centers.

In order to facilitate multi-center studies and utilize existing imaging data that can include a variety of CT scanners and scanning protocols, we sought to find a method to harmonize CT images of different scanning protocols for improving radiomics studies. Reconstruction kernel setting is one of the key confounding variables we can strive to control in radiomics to help us make correct and reproducible conclusions from our experiments [19,21,22]. Recently, Choe et al. [30] showed that a convolutional neural network (CNN) can convert CT image reconstruction kernels to reduce the effect of two different reconstruction kernels and improve the reproducibility of radiomic features in pulmonary nodules. The CNN uses deep learning to learn the differences between CT images of different resolutions, and then applies it on CT images to convert images of different kernels. They have made this CNN model publicly available for other researchers to apply to their research. However, this work was limited in that all the images came from one CT scanner with only two kernels (B30f and B50f), and their CNN model was not validated in a real-world clinical application.

In this study, we further fine-tuned this open-source CNN to convert reconstruction kernels of thin slice CT images. We then used the prediction of epidermal growth factor receptor (EGFR) status in lung cancer as an example, because lung cancer diagnosis and treatment are important topics of research, since various tumor characteristics have diagnostic and prognostic factors. For example, the treatment plan for lung adenocarcinoma has become tailored based on the tumor’s gene mutation status [10,31]. To determine tumor genotypes, molecular tests from tissue biopsies are considered to be the gold standard; however, biopsies are invasive and limited to a small sample of the tumor [32]. As a result, it is difficult to fully characterize the tumor’s spatial heterogeneity [33].

We show that CNN can create a more harmonized dataset from a randomized set of mixed reconstruction kernels, verified with an improvement in feature reproducibility and in EGFR prediction performance. Furthermore, we aim to select the best reconstruction kernel to set as the standard to maximize the reproducibility of the features and the EGFR prediction performance derived from the newly harmonized dataset. To our knowledge, this is the first study to utilize both the artificial intelligence (AI) kernel conversion method to harmonize image settings and the converted images to predict clinical information directly after the AI-aided harmonization.

## 2. Materials and Methods

### 2.1. Study Design

**Figure 1:**
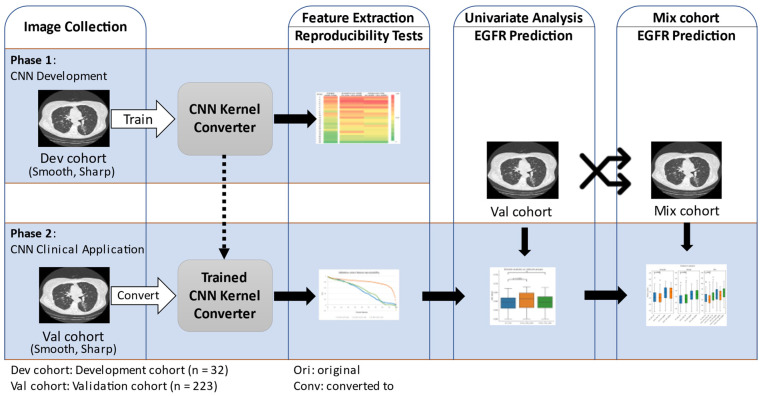
Study diagram. The diagram summarizes the two phases of our study.

The workflow for this study is shown in Figure 1. We first gathered CT images and created the development cohort and the validation cohort. The information on the patients and CT acquisition are described in the next subsection. The open-source CNN was trained using the development cohort to convert CT image reconstruction kernels from smooth to sharp and vice versa. The developed CNN kernel converter’s performance was assessed by testing the improvement in feature reproducibility after kernel conversion. The CNN kernel converter was then applied on the validation cohort, and its impact on improving radiomic feature reproducibility and predicting EGFR mutation status was analyzed.

### 2.2. Patient and CT Acquisition Info

The current study utilized deidentified CT images of non-small cell lung cancer (NSCLC) patients that were obtained and utilized for previously published studies [21,23]. The development cohort (16 men; mean age: 62.1 years; January–September 2007) was composed of 16,768 thin slice (1.25 mm) CT images of 32 NSCLC patients with 2 reconstruction kernel settings (Smooth: Standard; Sharp: Lung). It was part of the image data used in a previous publication [21]. The image series with the sharp kernel is available online and is known as The RIDER Lung CT [34]. The previous study was approved by the institutional review board, and it was Health Insurance Portability and Accountability Act (HIPPA) compliant.

**Figure 2:**
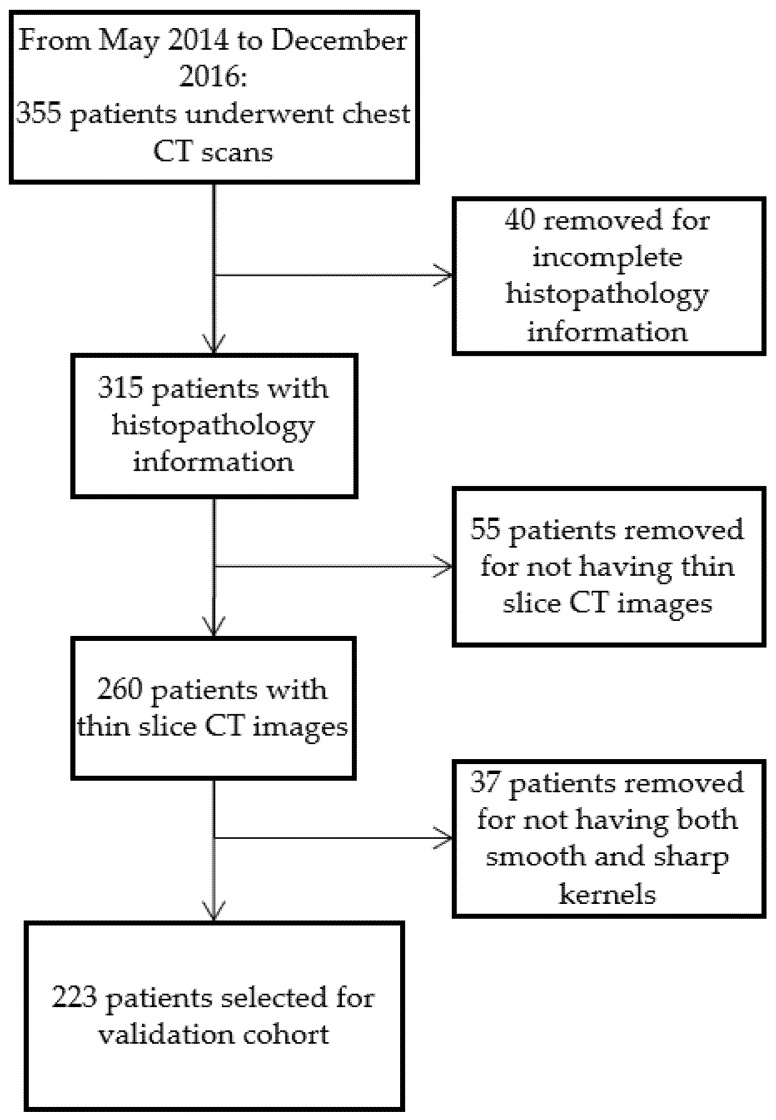
Flow chart of validation cohort patient selection process.

The validation cohort (127 men; mean age: 56.1 years; May 2014–December 2016) was composed of NSCLC patients of known EGFR statuses (114 EGFR/109 WT) with thin slice (1 mm) CT scans with different reconstruction kernel settings (smooth and sharp) and was retrospectively collected from the Second Xiangya Hospital of Central South University, China. A part of the cohort has been published before in Li’s study [23]. The institutional review board approved this retrospective study and waived the requirement for informed consent. The inclusion criteria were the following: (1) having completed molecular testing between May 2014 and December 2016, and (2) having underwent chest CT scans. The exclusion criteria were the following: (1) lack of complete histological and clinical information for the patient, (2) lack of thin slice CT scans, and (3) lack of both the smooth and sharp kernels. The process for patient selection is shown in Figure 2. Each patient had a molecular testing for EGFR status on the primary lung adenocarcinoma specimens from surgical resection or biopsy. The EGFR mutation status of the tumor was determined by utilizing an amplification refractory mutation system real-time technology using a human EGFR gene mutations fluorescence polymerase chain reaction diagnostic kit (Amoy Diagnostic Co., Ltd., Xiamen, China).

The CT imaging protocols used for the development cohort is found in Supplementary Materials Table S1 [21]. For the validation cohort, the CT scan acquisition parameters are shown in Supplementary Materials Table S2. General Electric (GE) (GE, Boston, MA, USA) CT scanners were used in the development cohort, and Siemens CT scanners (Siemens, Munich, Germany) were used in the validation cohort. Each CT scan was reconstructed into thin (1 mm for GE; 1.25 mm for Siemens) slice thickness with two reconstruction kernels (Smooth: B30f/B31s/B31f; Sharp: B60/B70s/B70f/B80). Each patient had two image sets labeled as “ori_smo” for the original images of smooth kernel and “ori_shp” for the original images of sharp kernel.

### 2.3. Lung Lesion Segmentation

Each patient had 1 lesion segmented in this study, for a total of 32 lesions for the development cohort and a total of 223 lesions for the validation cohort. Lesion segmentation for both cohorts was performed using a semi-automated watershed and active contours-based algorithm that is integrated into an image processing platform [35,36]. The segmentation for the development cohort was performed by three radiologists with 11, 10 and 25 years of experience interpreting oncologic CT images. The details of the segmentation and validation with inter-rate agreement can be found in the previously published paper [21]. For the validation cohort, the segmentation was performed by a radiologist with 20 years of experience (YL) on all images. To increase consistency, tumor segmentation was first performed on ori_shp images and then duplicated onto the ori_smo images. The radiologist was permitted to edit the duplicated contours if there were changes or shift of the segmentation on the images.

### 2.4. Radiomic Feature Extraction

For the development cohort, 89 fundamental features were extracted and analyzed to compare against the results from a prior experiment [19], which showed that there are differences in the concordance correlation coefficient (CCC) values caused by differences in reconstruction kernels. The 89 selected features were divided into 23 non-redundant feature groups, as previously done in order to replicate their results and to compare how the newly trained CNN kernel converter would affect the reproducibility of the feature groups. The features quantified tumor size, shape, boundary shape, tumor sharpness (e.g., sigmoid slope), histogram-derived density distribution, and texture patterns (e.g., gray-level co-occurrence matrix (GLCM), gray-level run-length matrix (GLRLM), gray-level size zone matrix (GLSZM), neighboring gray tone difference matrix (NGTDM), Laplacian of Gaussian, 3D Laws, and wavelet). The detailed description for the selection of the 89 features can be found in the Supplementary Materials Table S3 of the original manuscript [21]. For the validation cohort and its kernel converted counterparts, a total of 1158 features (composed of 89 previously mentioned features and their extensions) were calculated from the tumor region of interest (ROI). The in-house feature extractor and its 1158 features have been utilized and published in multiple articles [6,15,23,37,38,39]. Of note, our in-house feature extractor was developed prior to the IBSI (Image Biomarker Standardization Initiative) standard; we have compared our feature extractor with the IBSI [38] and showed that there were no significant differences in predicting EGFR mutation status in lung cancer when using either of the two feature extractors. Thus, we ultimately decided to use our in-house feature extractor because it is easy for us to perform analysis based on feature grouping due to the fact that we are more knowledgeable about our feature implementation.

### 2.5. CNN Kernel Converter Development and Validation

An open-source CNN [30] was re-trained using the development cohort to develop CNN models to convert the CT reconstruction kernels from smooth to sharp and vice-versa. Out of the 32 patients, 14 patients’ CT images were used to train two CNN models. There were 4628 images in the training set and 1560 images in the testing set to train the models. The learning rate was 1 × 10^−4^, the total number of epochs was 55 with each batch size at 2314, the optimization type was ADAM, and the loss function was sum of squares. The selection of our model’s training parameters was fine-tuned from the original paper [30] to better fit our model’s training and learning. The selection process was trial-and-error to minimize loss and converge the prediction error values between the training data and the testing data to prevent underfitting or overfitting, as is protocol with fine-tuning techniques. Furthermore, we set aside an additional 1012 images for quality check using root mean square error calculation between the output image generated by the CNN kernel converter and the ground truth (data not shown). Each training session took over 10 h. The newly trained networks for smooth to sharp conversion and vice-versa are uploaded and publicly available for use at the following GitHub page: https://github.com/jin-yoon34/CNN_kernel_conversion, and it can be applied using the method originally described by Choe et al. [30].

The implementation of CNN kernel converter is easy and quick. The CNN converter takes less than 0.5 s to generate 1 converted CT DICOM image. For each patient’s thin slice chest CT, it only takes a couple of minutes to convert the entire CT scan to another kernel using the CNN kernel converter. The total amount of time to generate new images varies due to each patient’s CT containing a varying number of images.

The features were extracted using our in-house feature extractor. The success of kernel conversion and feature reproducibility was confirmed by calculating the CCC [40] between the converted settings and the target settings for each feature. The CCCs ranged from −1 to 1, and a CCC value of 1 meant that there was a perfect correlation between the two calculated features.

The computer used in this study was an Intel Xeon Processor E5-2620 v4 2.1 GHz CPU with 128 GB DDR4 memory and an NVIDIA GeForce GTX TITAN Xp 12 GB GPU. The algorithms were implemented with Python 2.7 [41].

### 2.6. Randomization and Formation of Mixed Groups

To simulate a retrospective collection of images with varying reconstruction kernels, we created a mixed group, where we randomly assigned each of the 223 patients to either smooth or sharp kernels in order to create a mixed group to mimic multi-center data with heterogeneous reconstruction kernel settings. The resulting mixed group with the original images, “ori_mix”, was composed of 111 patients’ images with smooth kernels and 112 patients’ images with sharp kernels. Then, “conv_mix_smo” group was created by keeping all the patients with smooth kernels the same while converting all the patients with sharp kernels to smooth (conv_smo) using the CNN kernel converter, resulting in every patient in the group either having maintained its original smooth kernel or converted from sharp to smooth (conv_smo) kernel. Similarly, “conv_mix_shp” group was created by keeping all the patients with sharp kernels the same while converting all patients with smooth kernels to sharp (conv_shp) kernels.

### 2.7. Univariate Analysis

The effect of CNN kernel conversion on the mixture group was analyzed through univariate analyses. Univariate analysis was performed for each feature to predict the EGFR mutation status of the lung cancer. This analysis was performed in each kernel setting group including the three hypothetical mixture groups. The performance of each feature was measured using receiver operating characteristic (ROC) curve and area under the curve (AUC) of the ROC curve.

### 2.8. Statistical Analyses

Data are represented as mean ± standard deviation where appropriate. Statistical analyses were performed by using Python 3.8 [41]. To determine whether the kernel conversion affected the average CCC’s, we performed two-tailed Wilcoxon signed rank test before and after kernel conversion. The null hypothesis was that there was no difference between the medians of the CCC’s. *p* values less than 0.05 were considered significant. To determine whether the kernel conversion affected the results of univariate analysis, we performed two tailed Wilcoxon signed rank test before and after kernel conversion. To test whether there were differences between the wildtype (WT) groups and EGFR positive (EGFR) groups of varying kernel settings, we performed analysis of variance (ANOVA) to test for significance among the means of the groups being analyzed and multiple student’s *t*-tests for direct comparisons between two groups.

## 3. Results

### 3.1. Patient Demographics

A total of 255 NSCLC patients (development cohort: *n* = 32; validation cohort: *n* = 223) were included in the study, with each patient having CT images of both smooth and sharp kernels. The distributions of the validation cohort can be seen on Table 1. The validation cohort shows that there is no significantly different distribution between the WT and the EGFR groups in age, tumor stage or N-stage. There were significantly more males in the WT group than females, significantly more smokers in the WT group, more non-smokers in the EGFR group, and significantly more poorly-differentiated tumors in the WT group.

### 3.2. CNN Kernel Converter Development Using Development Cohort

An example of the CNN kernel conversion on CT images is shown in Supplementary Materials Figure S1. When given an input image of a specific kernel type (sharp in this example) to the CNN kernel converter network, it will be able to produce an output of the desired kernel (smooth in this example). Original smooth and sharp will be represented as ori_smo and ori_shp, respectively. A smooth image converted to sharp will be represented as conv_shp. The output (conv_smo) was compared against the ground truth (ori_smo) to measure the differences between the images. As seen in Supplementary Materials Figure S2, we observed a 99% decrease in root mean square error (RMSE) between the input image and ground truth image after the input image was converted to output image using the CNN kernel converter. The developed CNN network was applied on all 32 patients from the development cohort in a similar manner.

### 3.3. Effect of CNN Kernel Conversion on Radiomic Feature Reproducibility

#### 3.3.1. Development Cohort Radiomic Feature Reproducibility

**Figure 3:**
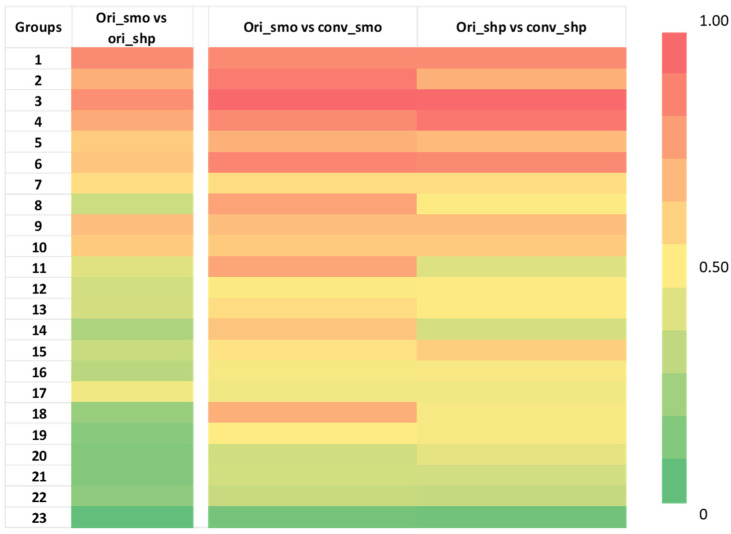
Heatmap of concordance correlation coefficient (CCC) of 87 radiomic features from development cohort divided into 23 groups as previously done for comparison [19]. Red represents a CCC value of 1, which means perfectly reproducible, while green represents a CCC value of 0, which means not reproducible. There is an increase in CCC after the kernel conversion in multiple feature groups. The names of the features within each group are indicated in Supplementary Materials Table 3. The numerical values can be found in Supplementary Materials Table S4.

We successfully converted all smooth and sharp kernel images using the CNN kernel converter to create two additional groups, conv_smooth and conv_sharp, and we successfully extracted 89 features (see Supplementary Materials Table S3) from all four image groups. As shown in Figure 3, the selected 89 features were divided into 23 features groups, and the results of three different comparisons are shown in a heatmap with red color showing the highest CCC at 1, and green color showing a low CCC value of 0. The average CCC increased after the kernel conversion for most of the feature groups in both conversions. Feature groups 1, 7, 9, 10, and 17, which are all shape features, did not have any changes in the average CCC after the kernel conversion in both groups. Only in comparison (ori_shp vs. conv_shp), feature groups 2 and 11 had small decreases in the average CCC by 0.003 and 0.008, respectively. Feature groups 18 and 19 had the highest increases in the average CCCs: for comparison (ori_smo vs. conv_smo), an increase of 0.632; for comparison (ori_shp vs. conv_shp), an increase of 0.581. Feature groups 18 and 19 were composed of the following texture features: Intensity_Skewness_2D, Intensity_Skewness_3D, GLCM_Entropy, GLCM_Diff_Entropy, Run_SPE, Run_PP, EdgeFreq_Mean, and LoG_Entropy_p1. The average and the median of the feature values all increased after the conversion. The Wilcoxon matched-pairs signed ranks test for (ori_smo vs. ori_shp) group against (ori_smo vs. conv_smo) group and (ori_smo vs. ori_shp) group against (ori_shp vs. conv_shp) group both showed *p* < 0.001, as seen in Table 2.

#### 3.3.2. Validation Cohort Radiomic Feature Reproducibility

**Figure 4:**
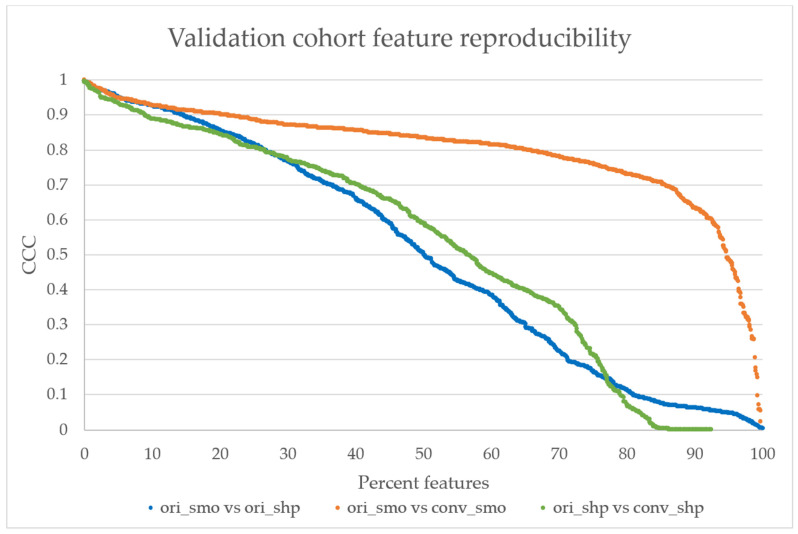
The validation cohort’s reproducibility graph. The reproducibility of features calculated from original smooth and sharp kernels show that only 20% of the features have CCC > 0.85 (highly reproducible) [21]. After the kernel conversion to smooth, more than 40% of features show CCC > 0.85, while only 20% of features from the sharp comparison show CCC > 0.85. The distributions between ori_smo vs. ori_shp and ori_smo vs. conv_smo are significantly different with *p* < 0.001, but the distributions are not significantly different between ori_smo vs. ori_shp and ori_shp vs. conv_shp.

All validation cohort images were successfully converted. The feature reproducibility calculations showed that there was a significant increase in the total number of features with CCC > 0.85 from 20% in the ori_smo vs ori_shp to 40% in ori_smo vs conv_smo, as seen in Figure 4. Table 3 shows that the average of all the CCC values also increased after kernel conversion to smooth with the original comparison at 0.50 ± 0.33 (average ± SD) to 0.80 ± 0.15 (*p* < 0.001). Median CCC is higher in ori_smo vs conv_smo than ori_smo vs ori_shp or ori_shp vs conv_shp.

### 3.4. Effect of CNN Kernel Conversion on EGFR Mutation Status Prediction

**Figure 5:**
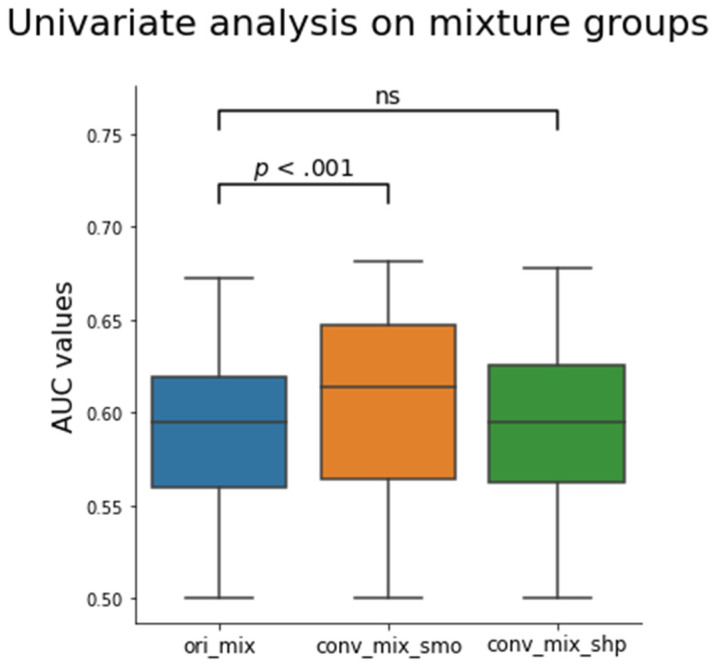
Box plot of the calculated AUC values for all 1158 features before and after the CNN kernel conversion on the mixture groups. Only the conv_mix_smo group was significantly different from the ori_mix group (Z = 15.1, *p* < 0.001). The median AUC values were 0.595 ± 0.006, 0.614 ± 0.028, and 0.595 ± 0.028 for ori_mix, conv_mix_smo, and conv_mix_shp groups, respectively. The Z values were 15.1 and 0.01 for conv_mix_smo and conv_mix_shp, respectively, when compared to the ori_mix group. The Z-values were calculated using two tailed Wilcoxon signed ranked test. ns = not significant.

**Figure 6:**
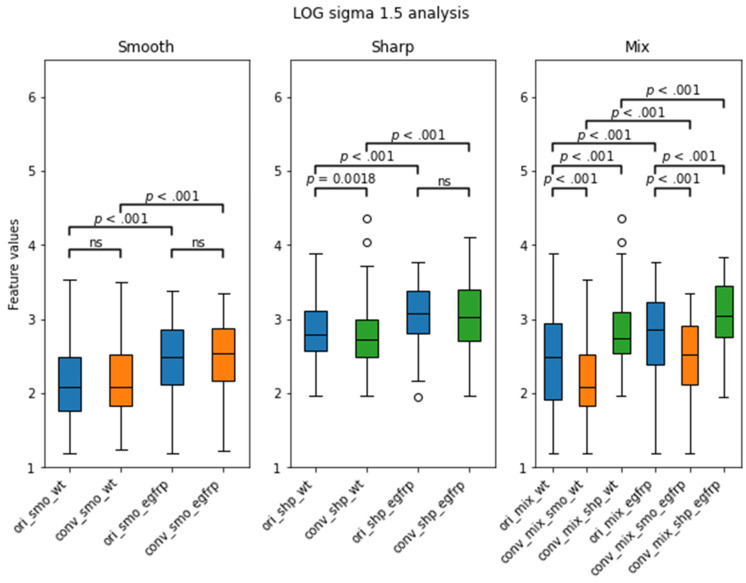
LOG sigma 1.5 feature’s distribution boxplot analysis from the validation cohort separated by EGFR status. WT stands for wildtype, and egfrp stands for EGFR positive. There were significant differences between the WT and the EGFR subgroups in all kernels. Notably, there was no significance between the original smooth subgroups and their converted smooth counterparts. *p* value less than 0.01 was considered to be significant. ns = not significant.

The distribution boxplot of the AUC values for each mixed setting is shown in Figure 5. The median for ori_mix was 0.595 ± 0.006 (median ± median absolute deviation) and the medians for conv_mix_smo and conv_mix_shp were 0.614 ± 0.028 and 0.595 ± 0.028, respectively. There was a significant increase in the median and distribution after the conversion to smooth (Z = 15.1, *p* < 0.001). There was no significant difference between the ori_mix AUC distribution and the conv_mix_shp AUC distribution (Z = 0.01, *p* = 0.49). Notably, the top three features with the highest AUC values that were selected for further analyses were texture-based features. There were two Laplacian of Gaussian features and one GLCM feature that showed improvement in CCC and EGFR status prediction after CNN kernel conversion, as shown in Table 4. In Figure 6, each subplot displays the boxplot for one of the top three selected texture features. In the non-mixed groups, the median AUC values of the converted images are shown to be similar to those of the originals for both the wildtypes and the EGFR positive types with the EGFR mutants having the higher median AUCs. In the mixture groups, kernel conversion maintained the separation of median AUCs and the similar pattern of EGFR positive mutants having the higher median AUC compared to the wildtypes.

## 4. Discussion

A method that can enable researchers to use a large collection of multi-setting CT images will be beneficial for improving the statistical power and clinical application of radiomic studies. Currently, many radiomic studies are limited due to having relatively small sample sizes and their lack of external dataset for validation, in part because these studies require a dataset of relatively homogeneous CT acquisition parameters [42,43,44,45], which may not have been available.

In this study, we successfully retrained a CNN model developed by Choe at el. [30] on one dataset and tested it on an external dataset acquired using a different CT scanner to convert the reconstruction kernels of CT images from smooth to sharp and vice versa. We then showed that kernel harmonization via a CNN converter can increase the reproducibility of radiomics features. There was an increase from 20 to 40 percent in the total number of features out of 1158 with CCC > 0.85 (which is considered to be highly reproducible) and an increase of 0.3 in the average CCC after the kernel conversion to smooth (*p* < 0.001). Furthermore, we observed an increase in the clinical predictive performance for predicting the EGFR mutation status of lung cancer lesions after the kernel conversion to smooth (median AUC = 0.614, Z = 15.1, *p* < 0.001).

With an increasing number of studies showing diagnostic and prognostic promise of radiomics in an era of personalized medicine [43,45,46], it is imperative that we improve the quality, reproducibility and robustness of radiomics research. Some critics have raised the concern that radiomic features are not robust and are susceptible to small differences in CT acquisition parameters [18,21,47]. The results of this study are consistent with previous studies on how CT reconstruction kernels affect radiomic feature values and reproducibility [48]. In the comparison between the two original kernel settings (ori_smo vs ori_shp), only 20% of 1158 features had CCC > 0.85. This susceptibility is a hindrance in radiomics studies and shows that datasets for radiomics studies cannot have heterogeneous kernel settings.

To address the non-biological impact of kernel setting on the radiomics results, Choe et al. [30] developed a CNN to convert the reconstruction kernels of retrospectively collected CT images acquired from Siemens and showed promising results in improving feature reproducibility. However, their group only trained their model using kernels B10f, B30f, B50f and B70f, and they did not have trained models for direct conversion between B30f and B70f, which are two of the most commonly used kernels in chest CT. In addition, the pretrained model’s kernel conversion performance was poor when used on the same-day repeat CT data acquired from GE. Thus, we retrained this open-source network for kernel conversion using the same-day repeat CT data (development cohort) [34] and successfully validated the CNN on an external dataset (validation cohort) that was acquired from Siemens.

Using the newly trained CNN kernel converter, we confirmed a similar improvement in the feature reproducibility using our in-house feature extractor. There was a significant improvement on average in the development cohort’s original CCC from 0.523 ± 0.314 to 0.763 ± 0.181 and 0.794 ± 0.178 for smooth and sharp conversions, respectively. Furthermore, it is worth noting that the newly trained CNN kernel converter was successful in converting the CT image data in the validation cohort, which had significantly different acquisition parameters from the development cohort used to train the network. Although we have split the image kernel groups to two simple groups (smooth and sharp), these groups actually contain a variety of algorithms. For instance, smooth group contains Standard/B30f/B31s/B31f, while sharp contains Lung/B60f/B70s/B70f/B80f. Our CNN that was trained on CT images from GE with 1.25 mm slice thickness and standard/lung kernels was successful in converting external CT images from Siemens with 1 mm slice thickness and a wide range of kernels (B30/B31f/B60f/B70f/B80f). This shows that our trained CNN does not require the input images to have exactly the same settings as the development cohort, and the CNN may be applied to CT images from other vendors with similar thin slices around 1 mm and similar kernel settings as smooth and sharp.

In our first phase of the experiment with the development cohort, we observed that certain feature groups increased in CCC more so than others after the kernel conversion. As seen in Figure 3, the CCC heatmap shows significant improvements in groups 18 and 19, which are composed of Intensity_Skewness_2D, Intensity_Skewness_3D, GLCM_Entropy, and GLCM_Diff_Entropy. These features currently show promise in the literature for EGFR prediction models as second order texture features that are highly predictive of EGFR mutation status: GLRLM, wavelet, LOG-sigma GLDM, LOG-sigma GLCM, skewness, short-run-low-grey-level-emphasis [49,50,51,52]. Many of these studies cite in their limitations that their homogeneous sample sizes are not large enough for machine learning or deep learning models. To increase the sample size for training and testing these prediction models, our trained CNN may be of use in harmonizing kernel settings to allow a larger dataset collection.

We applied the developed CNN to an external clinical CT data of lung cancer patients with known EGFR status. The CNN kernel harmonization improved the reproducibility of many features, as seen in Figure 4, with over 40% of 1158 features having high reproducibility at CCC > 0.85 after CNN kernel conversion to smooth kernel, which was a significant increase from 20% in the original set (*p* < 0.001). Harmonizing the image settings to sharp kernel did not improve in reproducibility, as the reproducibility calculation showed a similar percentage of features with CCC > 0.85 as the original smooth vs original sharp comparison at approximately 20% of the features (*p* > 0.05). The median CCC was also higher after the conversion to smooth kernel, but not for the conversion to sharp. Our results are in agreement with previous reports that show the smooth kernel having higher number of radiomics features with high reproducibility [19,20,23,30]. A possible reason for this might be that sharp reconstruction kernel, while it may provide higher resolution, also comes at the price of having images with significantly more noise.

Our results from the second phase show that conversion to smooth kernel may benefit clinical studies to predict EGFR mutation status. As previously mentioned, converting the kernel to smooth improved the reproducibility of the features. The univariate analysis results depicted in Figure 5 also show that there is a significant improvement in the median AUC in the conv_mix_smo group, with the median AUC increasing from 0.595 for the original mixture group to 0.614 (Z = 15.1, *p* < 0.001) in the converted smooth (sharp → smooth) mixture group. When we took a closer look at the top 3 features with the highest AUC values, as shown in Table 4 and Figure 6, we observe a significant improvement in CCC and a small improvement in AUC. The top three features were all texture-based features: two Laplacian of Gaussian (LOG) and one GLCM. LOG feature is an entropy-based quantification of image homogeneity with varying Gaussian filters. The top two Gaussian filters were filters with sigma of 1.5 and 2.5. GLCM is a histogram of co-occurring greyscale values at a given offset over an image to calculate how often pairs of pixels with specific values in a specific spatial relationship occur in an image. This finding is consistent with the first phase of the experiment and the literature. As previously mentioned, LOG and GLCM have been found to have clinical significance [49,50,51,52], especially in predicting EGFR. In our study, we have also found that these three texture features performed the best in predicting the EGFR status of the validation cohort, as measured by the AUC.

Some studies have proposed to approach the harmonization method in a statistical way, as has been accomplished in genomics using ComBat [26,27,53]. The advantages of the ComBat method are clear in that the method is easy, it can be performed on the given datasets without having to manipulate large image files, and it successfully harmonizes data statistically while accounting for various non-biological factors. However, one of the major disadvantages to ComBat is that it is difficult to set a standard to which to compare new data against, and any incoming new data cannot be adjusted on its own, requiring a set of data to harmonize the new data with. In the case of a CNN, any CT image may be given as an input, and there will be an output of converted images that can have its tumor features extracted and compared against a pre-set standard.

There are several limitations in our study. One limitation is that this study did not analyze individual lesion characteristics, so it is unclear if these individual characteristics have been harmonized. However, our goal of mutation status prediction was improved. Finally, our prediction model for the EGFR mutation status was a simple statistical analysis using the raw feature values for the univariate analysis. Univariate analyses are not comprehensive, and they are often utilized as the initial benchmark test to assess the feature’s potential in a more complex model. For instance, studies have shown that radiomic models using individual features perform worse than a multivariate model that uses machine learning or deep learning [23,42,49,52,54]. Further analyses with machine learning or deep learning models are needed to better assess how CNN kernel converter can improve feature reproducibility and clinical predictive performance.

## 5. Conclusions

Our study shows that the CNN kernel converter successfully improves the feature reproducibility and thus the performance of EGFR mutation status prediction after kernel harmonization in CT images. We also show that the better kernel for harmonization is the smooth kernel. The CNN kernel converter has promise for harmonizing CT images for improving multi-center or multi-setting radiomic studies of lung cancer.

## Figures and Tables

**Table 1:** Validation cohort patient characteristics.

	Wildtype (*n* = 109)	EGFR (*n* = 114)	*p* Value
Age (avg ± SD)	55.6 ± 10.6	56.6 ± 10.1	0.444
Sex			<0.001
Male	80	47	
Female	29	67	
Smoking status			<0.001
Smoking	54	30	
No smoking	55	84	
Stage			0.455
I	1	4	
II	5	4	
III	21	15	
IV	62	65	
Unknown	20	26	
N-Stage			0.541
N1	51	54	
N2	32	27	
Unknown	26	33	
Differentiation			<0.001
Low	72	38	
Well	32	66	
Unknown	5	10	

**Table 2:** Development cohort’s average and median reproducibility values calculated in CCC. The Wilcoxon matched-pairs signed ranks test results are shown for (ori_smo vs. ori_shp) group against (ori_smo vs. conv_smo) group and (ori_smo vs. ori_shp) group against (ori_shp vs. conv_shp) group.

	Ori_smo vs. Ori_shp	Ori_smo vs. Conv_smo	Ori_shp vs. Conv_shp
CCC (Avg ± SD)	0.523 ± 0.314	0.763 ± 0.181 *	0.794 ± 0.178 *
CCC (Median)	0.482	0.801	0.820
Wilcoxon W		0	3
*p* value		0.0002	0.0003

* Signifies *p* < 0.001.

**Table 3:** Validation cohort’s average and median reproducibility values calculated in CCC. The Wilcoxon matched-pairs signed ranks test results are shown for (ori_smo vs. ori_shp) group against (ori_smo vs. conv_smo) group and (ori_smo vs. ori_shp) group against (ori_shp vs. conv_shp) group.

	Ori_smo vs. Ori_shp	Ori_smo vs. Conv_smo	Ori_shp vs. Conv_shp
CCC (Avg ± SD)	0.499 ± 0.326	0.799 ± 0.149 *	0.515 ± 0.331
CCC (median)	0.504	0.835	0.589
*p* value		<0.001	0.17

* Signifies *p* < 0.001.

**Table 4:** The top three radiomic features with the highest AUC values compared among different mixed group settings.

	Reproducibility (CCC)			Prediction Performance (AUC)		
Feature Name	ori_smo vs. ori_shp	ori_smo vs. conv_smo	ori_shp vs. conv_shp	ori_mix	conv_mix_ smo	conv_mix_shp
Laplacian of Gaussian Sigma 2.5	0.888	0.922	0.961	0.672	0.679	0.676
Laplacian of Gaussian Sigma 1.5	0.445	0.941	0.891	0.641	0.681	0.669
GLCM	0.798	0.814	0.871	0.667	0.655	0.678

## Data Availability

Previously reported repeat CT image data were used to support this study and are available at [DOI: 10.7937/K9/TCIA.2015.U1X8A5NR]. Previously reported lung cancer patients of known EGFR statuses were used to support this study and request for data, after publication of this article, will be considered from the corresponding authors Lin Lu and Yajun Li at ll2860@cumc.columbia.edu and liyajun9966@csu.edu.cn. These prior studies (and datasets) are cited at relevant places within the text as references [25,27,33].

## Supplementary Materials

The following are available online at https://www.mdpi.com/article/10.3390/tomography7040074/s1, various supplemental figures and tables that are referenced in the main manuscript can be found in this separate Supplementary Materials file. They contain intermediate results or additional details of the cohorts or radiomics features. Figure S1: CNN kernel conversion example, Figure S2: Difference maps, Table S1: CT image scanner parameters for the development cohort, Table S2: CT image scanner parameters for the validation cohort, Table S3: A summary table for features groups analyzed in the development cohort, Table S4: CCC heatmap for the development cohort with CCC values.
