# Supplementary material for: Convolutional Neural Network Addresses the Confounding Impact of CT Reconstruction Kernels on Radiomics Studies
Source: Tomography. 2021 Dec 3;7(4):877–92. doi: 10.3390/tomography7040074 (PMC8707549; doi:10.3390/tomography7040074)
Supplement: Supplementary file 1 [file tomography-07-00074-s001.zip › tomography-1373683-supplementary.pdf]

Supplementary Materials

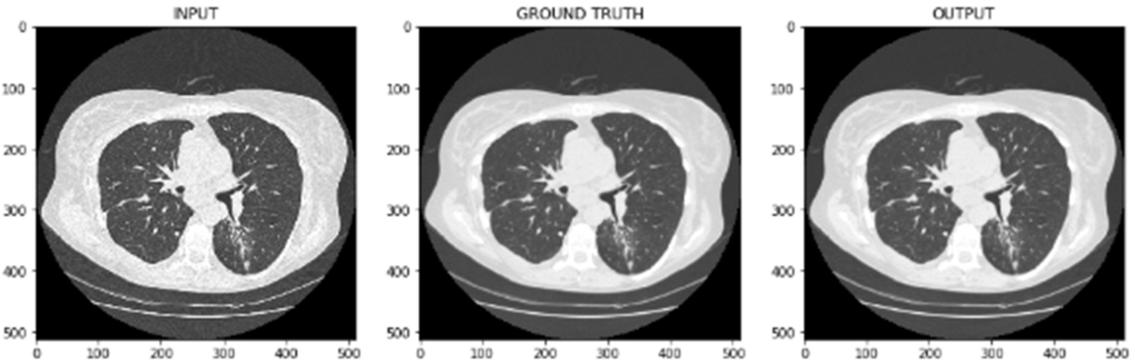

Figure S1. CNN kernel conversion example. An example of kernel conversion using the developed CNN network from sharp kernel (Input) to smooth kernel (Ground Truth). CT images of input (sharp), ground truth (smooth), and output (sharp -> conv_smooth) are shown, respectively.

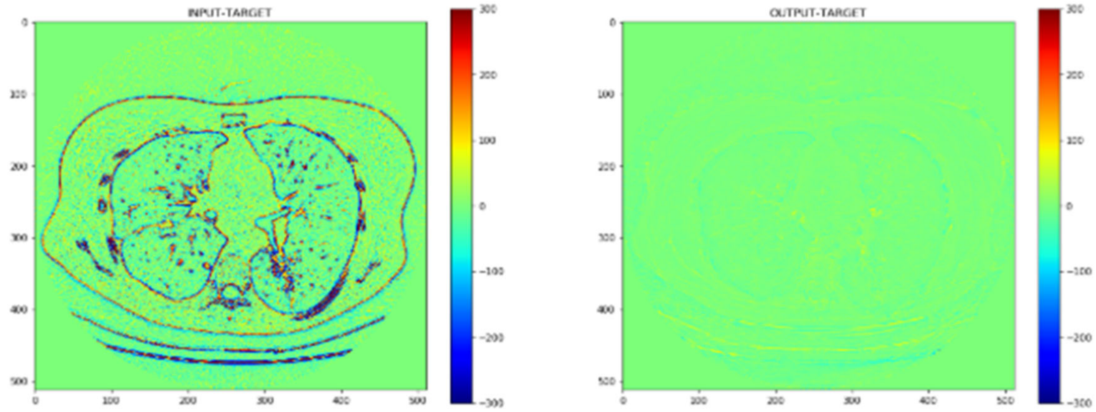

Figure S2. Difference maps. The difference maps are shown to show how much differences there are in the CT images between the input (sharp) and the target (smooth or ground truth) and between the output (conv_smo) and the target (smooth or ground truth). There was a 99% decrease in RMSE after CNN kernel conversion.

Table S1. CT image scanner parameters for the development cohort.

Scanning parameters	GE LightSpeed 16 (n = 28)	GE VCT (n = 4)
**Tube voltage**	120 kVp	120 kVp
**Tube current**	299-441 mA	298-351 mA
**Collimator configuration**	16 x 1.25 mm	64 x 0.63 mm
**Pitch**	1.375	0.984
**In-plane resolution**	0.51 x 0.51 – 0.90 x 0.90	0.50 x 0.50 – 0.87 x 0.87
**Slice thickness**	1.25 mm	1.25 mm
**Reconstruction algorithm**	Smooth: Standard<br>Sharp: Lung	Smooth: Standard<br>Sharp: Lung
**Contrast**	No	No

Table S2. CT image scanner parameters for the validation cohort.

Scanning parameters	Siemens Perspective (n = 86)	Siemens Sensation 64 (n = 50)	Siemens SOMATOM Definition AS (n = 12)	Siemens SOMATOM Definition Flash (n = 75)
**Tube voltage (kVp)**	110 and 130	120	120	120
**Tube current (mA)**	52 – 329	151 – 415	184 – 661	102 – 691
**Exposure (mAs)**	600	500	330 and 500	500
**Slice interval (mm)**	0.7, 0.9 and 1	0.8, 0.9 and 1	0.9 and 1	0.7, 0.8 and 1
**Slice thickness (mm)**	1	1	1	1
**Pixel spacing (mm)**	0.48 – 0.81	0.58 – 0.81	0.57 – 0.71	0.53 – 0.83
**Reconstruction algorithm**	Smooth: B31s<br>Sharp: B70s	Smooth: B30f and B31f<br>Sharp: B70f	Smooth: B31f<br>Sharp: B70f and B80f	Smooth: B30f and B31f<br>Sharp: B60f and B70f

Table S3. A summary table for features groups analyzed in the development cohort.

Group #	Feature Name(s)
**1**	Shape_Index_8
**2**	GTDM_Strength, GTDM_Coarseness, EdgeFreq_Coarseness
**3**	Sigmoid_Offset_Mean
**4**	Shape_Index_2, Shape_Index_3, Shape_Index_4, Shape_Index_5, Run_PLU, Run_GLU, Uni_2D, Bi_2D, Vol, Uniformity_p4
**5**	LoG_Entropy_p4
**6**	Sigmoid_Amplitude_Mean, Intensity_Mean_2D, Intensity_Mean_3D, GLCM_Sum_Average, GLCM_Sum_Variance
**7**	Eccentricity
**8**	Spatial
**9**	Shape_Index_9
**10**	Solidity, Compact_Factor, Round_Factor
**11**	LoG_MGI_p1, LoG_MGI_p4
**12**	DWT_LH, DWT_H, Gabor_dir90
**13**	Intensity_Std_2D, Intensity_Std_3D, Laws_1, GLCM_Contrast, GLCM_Sum_Squares, Cluster, Laws_2, Laws_3, Laws_5, Laws_6, Laws_8, Laws_11, Laws_12
**14**	Intensity_Kurtosis_3D, Intensity_Kurtosis_2D
**15**	EdgeFreq_Contrast, GTDM_Contrast
**16**	Gabor_dir0, DWT_V, DWT_LV, DWT_LD, Gabor_dir45, DWT_D, Gabor_dir135, Gabor_sum
**17**	Shape_Index_6, Shape_Index_7
**18**	Intensity_Skewness_2D, Intensity_Skewness_3D, GLCM_Entropy, GLCM_Diff_Entropy, Run_SPE, Run_PP, EdgeFreq_Mean, LoG_Entropy_p1
**19**	Run_LPE
**20**	GLCM_IMC1, Fractal
**21**	GLCM_IMC2, GLCM_Corr, GLCM_MCC
**22**	Laws_10, Laws_4, Laws_7, Laws_9, Laws_13, Laws_14, GTDM_Complexity
**23**	Sigmoid_Slope

Table S4. CCC heatmap for the development cohort with CCC values.

Groups	a) Original smooth vs sharp	b) smooth vs conv_smooth (ori_sharp -> conv_smooth)	c) sharp vs conv_sharp (ori_smooth -> conv_smooth)
1	0.928	0.928	0.928
2	0.876	0.950	0.873
3	0.922	0.977	0.975
4	0.884	0.928	0.957
5	0.833	0.873	0.859
6	0.843	0.935	0.930
7	0.811	0.811	0.811
8	0.515	0.893	0.788
9	0.856	0.856	0.856
10	0.835	0.835	0.835
11	0.624	0.889	0.616
12	0.552	0.770	0.780
13	0.562	0.811	0.780
14	0.361	0.846	0.568
15	0.503	0.802	0.832
16	0.425	0.741	0.749
17	0.717	0.717	0.717
18	0.245	0.876	0.744
19	0.162	0.788	0.744
20	0.128	0.541	0.656
21	0.135	0.556	0.553
22	0.191	0.492	0.481
23	-0.040	0.068	0.049
